# *Morinda citrifolia* (Noni) as an Anti-Inflammatory Treatment in Women with Primary Dysmenorrhoea: A Randomised Double-Blind Placebo-Controlled Trial

**DOI:** 10.1155/2013/195454

**Published:** 2013-01-29

**Authors:** H. M. Fletcher, J. Dawkins, C. Rattray, G. Wharfe, M. Reid, G. Gordon-Strachan

**Affiliations:** ^1^Department of Obstetrics and Gynaecology, The University of the West Indies, Mona, Kingston 7, Jamaica; ^2^Department of Pathology, The University of the West Indies, Mona, Kingston 7, Jamaica; ^3^Tropical Metabolism Research Unit, The University of the West Indies, Mona, Kingston 7, Jamaica; ^4^Faculty of Medical Sciences, The University of the West Indies, Deans Office, Mona, Kingston 7, Jamaica

## Abstract

*Introduction*. Noni (*Morinda citrifolia*) has been used for many years as an anti-inflammatory agent. We tested the efficacy of Noni in women with dysmenorrhea. *Method*. We did a prospective randomized double-blind placebo-controlled trial in 100 university students of 18 years and older over three menstrual cycles. Patients were invited to participate and randomly assigned to receive 400 mg Noni capsules or placebo. They were assessed for baseline demographic variables such as age, parity, and BMI. They were also assessed before and after treatment, for pain, menstrual blood loss, and laboratory variables: ESR, hemoglobin, and packed cell volume. *Results*. Of the 1027 women screened, 100 eligible women were randomized. Of the women completing the study, 42 women were randomized to Noni and 38 to placebo. There were no significant differences in any of the variables at randomization. There were also no significant differences in mean bleeding score or pain score at randomization. Both bleeding and pain scores gradually improved in both groups as the women were observed over three menstrual cycles; however, the improvement was not significantly different in the Noni group when compared to the controls. *Conclusion*. Noni did not show a reduction in menstrual pain or bleeding when compared to placebo.

## 1. Introduction

Dysmenorrhoea, or painful menstruation, is a common gynaecological problem that affects adolescents and women of reproductive age and can cause severe disability. It is the leading cause of recurrent school absence in adolescent girls [1]. Risk factors for dysmenorrhea include nulliparity, heavy menstrual flow, smoking, and depression [1]. Primary dysmenorrhoea occurs in the absence of identifiable pelvic pathology, whereas secondary dysmenorrhoea occurs in the presence of a pelvic pathology, such as endometriosis, adenomyosis, uterine leiomyomata, or chronic pelvic inflammatory disease [2]. 

Empiric treatment for primary dysmenorrhoea may be initiated based on a typical history of painful menstruation, in addition to a negative clinical examination. The pain of dysmenorrhoea is believed to be mediated by the release of prostaglandin F2 alpha (PGF2*α*) in menstrual fluid [3, 4], and the suppression of prostaglandin as a treatment for dysmenorrhoea has become standard treatment [5]. The treatment of choice for initial management is nonsteroidal anti-inflammatory drugs (NSAIDs) in patients suspected to have primary dysmenorrhoea [2]. Other treatment options include oral contraceptives and depo medroxyprogesterone acetate. Patients who do not experience sufficient pain relief may be treated by prolonged cyclical oral contraceptive pills. Some patients do not desire hormonal contraception but desire alternative remedies. Topical heat, vitamin E, fish oil supplements, acupressure, low-fat vegetarian diet, and Japanese toki-shakuyaku-san have shown some benefit [1]. 

*Morinda citrifolia *(Noni) is a tree in the coffee family: Rubiaceae. Its native range extends through Southeast Asia and Australasia, and it has been used extensively in folk medicine by Polynesians for over 2000 years and has been reported to have a wide range of therapeutic benefits [6], including those of anti-inflammatory [7], immunomodulatory [8], and gastrointestinal [9]. Reports on the preventative effects of Noni on cancer, infection, arthritis, diabetes, and pain have also been published [6, 10]. 

Noni is used commonly by Jamaicans especially in rural districts. The fruit is prepared in many ways, and the juice and capsules are available commercially in many pharmacies. The analgesic effect of Noni is due to its ability to inhibit cyclooxygenase and is as potent as some NSAIDs [11]. It also has tranquilizing properties, similar to narcotic agents [7]. It is believed to be nonaddictive and side-effect-free. 

The study was a randomized double-blinded control study designed to evaluate the effect of 400 mg of Noni twice daily compared with placebo on pain and menstrual blood loss in the treatment of primary dysmenorrhoea. The duration of the study in each subject was three months, including a month for baseline analysis of the subjects' cycle. 

The study as performed was a superiority trial. We wanted to test the hypothesis that Noni was superior to placebo in reducing dysmenorrhea. Dysmenorrhea was measured in this study by visual analog pain scores, and bleeding was measured using pictorial charts. Both of these were regarded as primary endpoints, while haemoglobin and packed cell volume (PCV) or hematocrit and erythrocyte sedimentation rate were secondary end-points. The design of the trial was a repeated measures design with 1 baseline and 2 posttreatment measurements. A sample size of 40 in each group would allow us to detect a 0.5 standard deviation difference in pain scores between Noni and placebo assuming a correlation between baseline and posttreatment measurements of 0.7 at 90% power and an alpha of 0.05.

## 2. Methods

Young women aged 18 years and older attending any of the major educational colleges in the Kingston metropolitan area who were suffering with dysmenorrhoea were invited to participate in the study, and informed consent was obtained. Subjects were advised of the three month follow-up period, and daily tally of the sanitary napkins and tampons used. All subjects were interviewed to determine demographic data, age, parity and use of over the counter or prescription analgesics. Those with a history of previous surgery, pelvic surgery, hypersensitivity to Noni, liver disease, and suspected pregnancy were excluded. A total of 100 subjects were enrolled into the study. 

An initial examination was done to determine height and weight, and a baseline complete blood count and erythrocyte sedimentation rate were done. Each subject was given a visual analog pain score to assess the severity of the pain [12] with 0 being no pain and 10 being the worst pain the subject ever experienced. Patients were asked to refrain from the use of other treatment options for dysmenorrhoea during the study period. All of participants admitted to the use of analgesic agents for dysmenorrhoea prior to enrolment in the study.

The blood loss was measured with a pictorial blood loss assessment chart (PBLC) [13] provided to each participant. This chart consisted of diagrams depicting saturation of sanitary pads: minimal, moderate, and maximal. The subjects were required to document each pad removed during the menses by making a tally mark in the appropriate day of the respective cycle. The stained pads were given the score of 1 for minimal staining, 5 for moderate staining, and 20 for maximal staining. Clots were also assessed: a small clot (10 cent coin) was one point, and the large one was given 5 points (10 dollar coin). Flooding was defined as a gush of blood that may or may not overflow the pad and was also given 5 points. 

The Noni capsules were purchased from Vitamin World in Ronkonkoma, New York, USA. Each capsule contained calcium sulphate, gelatin, silica, vegetable magnesium stearate, and 400 mg of pure milled Noni herb powder. The placebo was obtained by purchasing empty gelatin capsules (Capsuline) similar in size shape and colour to the Noni capsules. These were filled in the pharmacy department by a pharmacist with glucose. Each container was randomly numbered using a computer-generated table of random numbers and each set of the capsules, Noni, or placebo was placed in identical containers and placed in sealed numbered packets. A total of twenty capsules were placed in each packet. The study was double blinded, and the numbers applying to the content of each packet were sealed and unknown to the subjects and primary researcher until the completion of the study. Subjects were advised to start the capsules in the second menstrual cycle of the study. The dosage was one tablet twice daily for five days, beginning 2 days prior to the onset of the menses and half an hour prior to meals.

Subjects randomly received a numbered packet, which was used as their study number and a folder containing a file for each of the three months of the study. Each file contained a visual analog pain score (VAS) and PBLC labeled with the subject's study number and the number of the cycle (1, 2, and 3).

**Figure 1:**
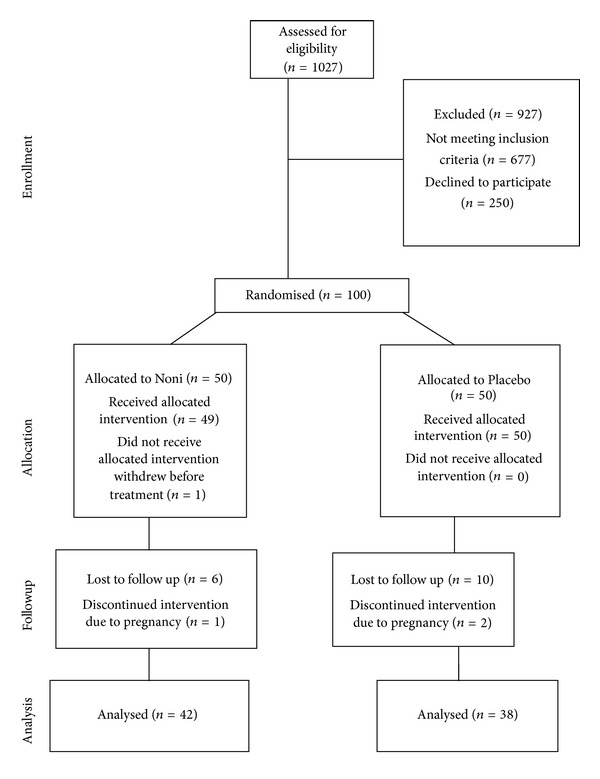
Participant flow diagram.

The data collected was the worst pain score daily and the maximum and minimum score for each cycle. The median pain score for each cycle was calculated at the end of the study. The bleeding score was tallied for each cycle, and the difference in the bleeding score was recorded. The haemoglobin, packed cell volume (PCV) or hematocrit and erythrocyte sedimentation rate (ESR), were all repeated at the end of the study (Figure 1). 

## 3. Statistical Analysis

Values are presented as means with standard deviation (SD), medians with interquartile ranges, or counts as appropriate. For continuous outcome variables with normal distribution differences in means by categories were tested with analysis of variance (ANOVA) or *t*-test as appropriate. For continuous outcome variables that were skewed, differences in distribution by categories were tested with Kruskal-Wallis test or the Mann-Whitney test.

For categorical outcome variables, differences were tested with chi square. The binomial test was used to compare the observed proportion in this study with expected proportion in the general population.

The study was approved by the UHWI ethics committee, and all patients provided written consent.

## 4. Results

A total of 1027 students were approached. Following screening, 350 were eligible, 250 refused to participate, and 100 were enrolled. There were 50 subjects in each arm. Three subjects became pregnant and were excluded. An additional three were started on continuous oral contraceptive pills and did not complete the study, and an additional 14 were lost to follow up. The final analysis was conducted on 80 subjects. The allocation of patients excluded from the analysis was 12 for the placebo arm, and 8 from the Noni arm.

The covariates that were analyzed at randomization were age, body mass index (BMI), haemoglobin, ESR, maximum, minimum and median pain score, and mean bleeding score. 

The mean age at randomization for the Noni group was 22.7 years and 22.0 for the placebo group; the difference in the means was not statistically significant. There were no significant differences in any variable between the two groups at randomization (Table 1(a) and 1(b)). The majority (88%) of patients were nulliparous. Seven percent of subjects were para 1, 3% para 2, and 2% para 3.

The mean haemoglobin at randomization was 11.7 g/dL for placebo, and 11.5 g/dL for Noni. The mean haemoglobin after the completion of the study was 11.75 g/dL for placebo, and 11.74 g/dL for Noni, which was not statistically significantly different (*P* value = 0.6). 

The mean ESR at randomization was 15.29 mm/s for placebo, and 18.94 mm/s for Noni, and the ESR after the study was completed was 13.47 mm/for placebo and 10.56 for Noni. The difference in the means of the ESR between start and end of the study between the two groups was statistically significant (Table 2(a)) with the controls having a higher ESR at completion.

The mean bleeding score at baseline was similar in both groups at 88.42 (SD 54.76) and 99.30 (SD 70.69) for placebo and Noni, respectively. At the end of the third cycle, the mean bleeding score was 70.93 for placebo and 87.70 for Noni, with a bleeding score difference from baseline to the third cycle of 4.18 (SD 43.7) for placebo, and 7.19 (SD 30.41) for Noni. When this difference was analyzed with the *t* test, the *P* value was 0.405, and, as such, the difference in mean bleeding score between the two groups was not statistically significant (Table 2(b)).

**Figure 2:**
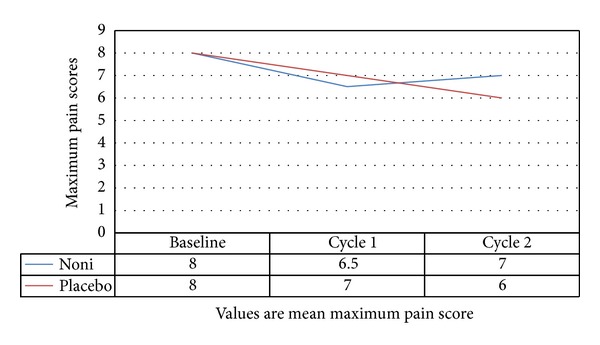
Comparison of the effects of noni versus placebo on pain score.

In both groups, the mean maximal pain at baseline was similar: 8.00 (SD 2.10) for placebo and 8.02 (SD 2.18) for Noni. At the end of the second cycle, there was a marginal decrease in the mean maximal pain in the Noni group compared to the placebo group with pain score of 6.59 (SD 2.70) and 7 (SD 2.90), respectively. At the end of the third cycle, the placebo group had more of a decrease in mean pain scores compared to Noni group, with values of 6.24 (SD 3.11) and 7.02 (SD 2.54), respectively, (Figure 2 and Table 3). 

Twenty one patients, 13 from the placebo and 8 from the Noni group, had previously used the Noni extract for dysmenorrhoea, with what they all said was with good effect.

## 5. Discussion

It has been estimated that up to 72% of young women experience dysmenorrhoea [14], and that 15% have such severe dysmenorrhoea that it affects their quality of life [14, 15]. The pain of dysmenorrhoea is believed to be mediated by the release of prostaglandin F2 alpha (PGF2*α*) in menstrual fluid [3, 4], and the suppression of prostaglandin as treatment for dysmenorrhoea has become standard treatment [5]. Vasopressin is released from the posterior pituitary, is found in higher concentrations in patients with dysmenorrhea [16], and is thought to increase uterine contractility, thereby resulting in ischaemic pain [17, 18]. Substances that block these vasopressin receptors are associated with decreased dysmenorrhoea but are not used routinely at present.

The standard mode of treatment for dysmenorrhoea involves the use of nonsteroidal anti-inflammatory drugs (NSAIDs), or combined oral contraceptive pills [19–21]. NSAIDs are very effective in many patients, but have relative contraindications such as risk of gastritis and upper gastrointestinal bleeding with long-term use. Combined oral contraceptive pills decrease endometrial proliferation, and thus prostaglandin synthesis at the level of the endometrium, decreasing menstrual bleeding and dysmenorrhoea. The use of combined oral contraceptive pills in the adolescent age group is not always culturally accepted. 

Noni has been said to possess analgesic and sedative effects that are nonaddictive [7, 11, 22]. The analgesic effects are believed to be as strong as morphine [7], and no side effects have been demonstrated [23, 24]. A previous animal study on Noni done in Jamaica did demonstrate its anti-inflammatory efficacy in lab-induced arthritis in rats [25]. It is therefore believed to have a better side effect profile compared with other drugs. 

The fact that the subjects using Noni had a better decrease in their ESR (an acute phase reactant) suggests that it does have some anti-inflammatory effects; however, this was not manifested in clinically improved pain or bleeding scores during the time of the study.

Previous studies have demonstrated a relationship between the severity of dysmenorrhoea and the amount of menstrual blood loss [14]. The analysis of the study revealed that Noni use was not associated with a reduction in the severity of pain from primary dysmenorrhoea, nor the amount of menstrual blood loss compared with placebo. 

The majority of patients expressed dysmenorrhoea that was worse on the first day of the cycle. This was expressed as the maximal pain score. The mean maximal pain score was similar between groups throughout the study period, and although there were minor reductions among the groups in the baseline, second, and third cycles, this was not statistically or clinically significant. 

A limitation in the study was a high refusal rate of 71.4% at recruitment. This may have possibly affected the reproducibility of and introduced bias into the study as the patients who participated were likely to have more severe dysmenorrhoea. 

Another limitation of the study was that it was not possible to verify that the subjects were compliant with the dosage of the Noni, starting at two days before the onset of menses. The follow-up period was also long (3 months from recruitment), and this may have led to compliance issues. We did not analyze for the effect of previous treatment of dysmenorrhoea and its effect on the baseline data. The follow-up period of 12 weeks was twice as long as the six-to-eight weeks recommended by some manufacturers who claim that it works slowly, and this long period is needed before an effect is seen (Drug insert Vitamin World).

The dosage used in the study was 400 mg twice daily for five days, for ease of administration. The dosage of Noni in extracts that have been said to be subjectively useful was 500 mg, and there are different preparations available that have been used in other studies (Noni Juice). Studies assessing for the toxic levels of Noni in mice have shown no adverse effects [11], and a higher dose may be required for a significant effect to be seen. 

Only 80 of 100 patients were analyzed, 38 in the placebo arm and 42 in the Noni arm. Using this small sample size the decrease in pain score appears to be a placebo effect. Further studies using higher doses of Noni with a larger sample size are needed.

## Figures and Tables

**Table:** (a)

Variable	Placebo	Noni	*P* value
Age (years)	22 ± 3.9	22.7 ± 5.4	0.4
Weight (kg)	60.7 ± 11.1	64 ± 11.1	0.1
BMI (kg/m^2^)	22.8 ± 3.9	24.3 ± 4.2	0.07
Hb (g/dL)	11.7 ± 1.4	11.5 ± 1.3	0.6
PCV (%)	36.4 ± 3.5	35.9 ± 3.4	0.5

Values are means ± SD or median with minimum and maximum.

**Table:** (b)

Variable	Placebo	Noni	*P* value
ESR	12 (0,75)	13 (4,68)	0.1
Parity	0 (0,3)	0 (0,3)	1.0
Maximum pain	8 (3,10)	9 (3,10)	0.9
Minimum pain	0 (0,3)	0 (0,6)	0.6
Median pain	5 (1,9)	5 (2,8)	0.3
Blood loss score	72.5 (21,198)	82.5 (11,249)	0.6

Values are median with minimum and maximum.

**Table:** (a)

Variable	Mean difference placebo versus Noni (95% CI)	*P* value
Haemoglobin (g/dL)	−0.08 (−0.41 to 0.26)	0.6
Packed cell volume	0.005 (−1.21 to 1.22)	0.9
ESR (mm/sec)	0.79 (0.6 to 0.9)	<0.02

**Table:** (b)

Variables	Placebo	Noni
	Mean (SD)	Mean (SD)
Hb before study (g/dL)	11.68 (1.39)	11.52 (1.33)
Hb after study (g/dL)	11.75 (1.10)	11.74 (1.01)
Hb difference (g/dL)	0.07 (0.85)	0.22 (0.78)
ESR before study (mm/sec)	15.29 (13.07)	18.94 (14.46)
ESR after study (mm/sec)	13.47 (9.82)	10.56 (6.66)
Bleeding score before	88.42 (54.76)	99.31 (70.69)
Bleeding score after second cycle	85.00 (50.37)	91.43 (70.37)
Bleeding score after third cycle	70.93 (54.39)	87.70 (59.70)
Bleeding score difference	4.18 (43.72)	7.19 (30.41)

**Table 3:** Comparison of the effects of Noni versus placebo on pain score.

Variable	Placebo		Noni	
	Mean (SD)	95% confidence interval	Mean (SD)	95% confidence interval
Maximum pain score baseline	8.0 (2.1)	7.30–8.69	8.0 (2.1)	7.34–8.70
Maximum pain score after second cycle	7.0 (2.9)	6.04–7.45	6.5 (2.7)	5.75–6.43
Maximum pain score after third cycle	6.0 (3.1)	5.05–7.42	7.0 (2.5)	6.17–7.83
